# Disposable Foamed Silicone Composite Actuator Powered by Sublimation

**DOI:** 10.3390/polym17223032

**Published:** 2025-11-15

**Authors:** Igor Bezsudnov, Alina Khmelnitskaia, Aleksandra Kalinina, Sergey Ponomarenko

**Affiliations:** Enikolopov Institute of Synthetic Polymeric Materials of Russian Academy of Sciences (ISPM RAS), Profsoyuznaya Str. 70, 117393 Moscow, Russia; alina.khmelnitskaya@ispm.ru (A.K.); kalinina@ispm.ru (A.K.); ponomarenko@ispm.ru (S.P.)

**Keywords:** soft actuator, silicone foam, working liquid, sublimation, rejuvenation procedure, open-air performance

## Abstract

Soft actuators are widely explored as movers in various devices, human–machine interfaces, for medical purposes and other biomedical applications. Among them are soft actuators based on a foamed silicone matrix with the working liquid (WL) captured in its pores that undergo the liquid–gas phase transition. For the first time, to gain the actuation strain of such composites, we added, to the WL, a substance that sublimates during the composite actuation. C1–C3 alcohols were tested as WLs, while the sublimation substance (SS) used was benzoic acid dissolved in the WL. It was found that the rejuvenation procedure is able to fill the composite pores with WL + SS solution. The effect of benzoic acid addition was revealed using the two-stage heating mode. The sublimation substance effectively extends the composite strain for methanol and ethanol as WL for about 20%. For C3 propanols, the strain is left nearly unchanged. In the open-air conditions, the high diffusion of WL + SS in silicone allows only a single actuation that makes it a disposable actuator, i.e., a kind of safety switch is proposed. The results obtained in this work pave the way to future, powerful multipurpose “soft safeties” appliances.

## 1. Introduction

The significance of understanding the operation of soft actuators is difficult to overestimate; they are increasingly used in common life as movers or actuators in various devices, in human–machine interfaces, for medical purposes, etc. To date, many reviews have been published that describe the use of different actuator types [1,2,3], including specific applications, for instance, in biomedicine [4,5,6], etc.

By now, there is also a wide range of reviews on virtually every kind of actuator, particularly polymer ones. Thus, ionic polymer–metal composites are described in detail in [7,8], and ionic polymer gels [9,10], conductive polymers [11,12], polymer electrets [13,14], ferroelectric polymers [15,16], liquid crystal elastomers [17,18], and dielectric elastomer actuators are described in detail and generally in [19,20,21,22]. Of course, the actuator types are not limited to those listed above and new ones are constantly emerging, adapted to specific operating conditions or performing specific tasks.

A foamed polydimethylsiloxane (PDMS) matrix with closed, hollow spherical inclusions is the framework for a composite using any type of phase transition. Thus, if the pores of the matrix are filled with a solid substance that, under heating, undergoes a solid–liquid phase transition with volume expansion, the actuation can be observed. The operation of such type of actuators based on paraffin was demonstrated in [23,24].

Recently, a new kind of soft actuators has been proposed [25,26] with the pores filled with a liquid utilizing a liquid–gas phase transition. The volume change during the liquid–gas phase transition is significantly higher (>20%) than for the solid–liquid paraffin transition (approx. 3%). The silicone composite matrix with closed spherical pores containing ethanol was proposed in [26] as “a single easily prepared soft robust material that combines the elastic properties of a polymeric matrix and the extreme volume change of a fluid upon liquid–vapor transition”.

A number of works were devoted to the operation optimization of these devices. Thus, an important result on the method of restoring their functioning after actuation–rejuvenation [27] has been obtained, and various heating techniques [28,29,30] and methods of increasing thermal conductivity [31], allowing us to accelerate the process of actuation, have been proposed, and the possibility of using alcohols C1–C4 for actuation has been investigated [32]. It is also worth noting the possibility of additive manufacturing for this type of actuator [33].

This study describes the investigation of the foamed silicone composite actuators applying sublimation substance power as the actuation buster. We use the rejuvenation procedure [27] by applying alcohols C1–C3 as a working liquid (WL) [32] with a sublimation substance (SS)—benzoic acid dissolved in WL. During actuation, the WL undergo a liquid–gas phase transition and the SS sublimes, powering the strain of the actuator.

The reasons for selecting benzoic acid as the sublimation substance were the following: its high vapor pressure at actuation temperatures, relatively good solubility in the WL used in our experiments, i.e., C1–C3 alcohols, as well as high diffusion coefficients of its alcohol solutions in the silicone matrix. The last two features allow the maximum possible amount of SS to be delivered to the pores of the composite. Also, sufficiently high boiling and decomposition temperatures of the selected substance to be converted into vapor upon heating were taken into account. In addition, according to environmental considerations, the substance has to be non-toxic and environmentally friendly. Naphthalene, iodine, and benzoic acid were considered as possible candidates for a sublimation substance. Despite the excellent saturated vapor pressures of naphthalene (1160 Pa @ 25 °C) and iodine (~100 Pa @ 25 °C) at room temperature, their use was impractical due to the toxicity and unpleasant odor of naphthalene and the inconvenience of iodine handling. Thus, we have chosen benzoic acid as SS for this work. The additional advantages of benzoic acid were the following: its active sublimation begins only at 90–100 °C, i.e., significantly above the room temperature, it is a food additive E210, and also it is environmentally friendly.

The experiments were carried out in the open air, and that makes such actuators of the disposal type.

## 2. Materials and Methods

### 2.1. Materials

Ecoflex 00-50 (Smooth-On, Macungie, PA, USA)—a two-part Pt catalyzed silicone—is a composite matrix. The ethanol was used as a pore-forming agent (PFA) [32], the working liquid (WL) is either ethanol (96%) or methanol (99.5%) or isopropanol (99.5%) or propanol (99.5%). All alcohols were reagent grade (EKOS-1, Moscow, Russia). The benzoic acid (99.5%), (EKOS-1, Moscow, Russia) was used within the experiments as the sublimation substance.

### 2.2. Foamed Silicone Preparation

**Figure 1:**
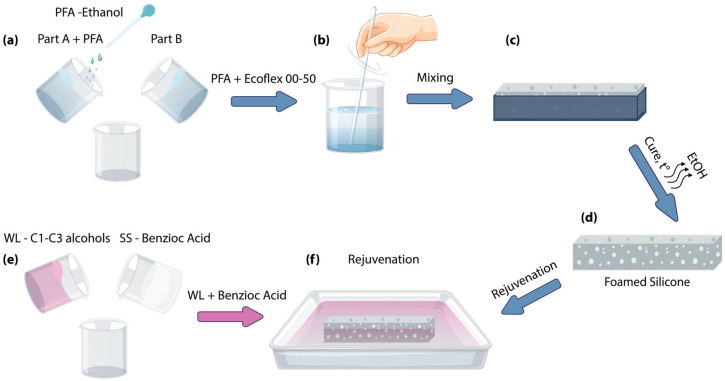
Sample preparation: (**a**) mixing Ecoflex 00-50 component A with ethanol and adding component B; (**b**) mixing all components; (**c**) the mold with the sample; (**d**) ready foamed composite; (**e**) dissolving sublimation substance in the working liquid; and (**f**) the composite rejuvenation.

Figure 1 schematically shows the sequence of the sample preparation. First, on the base of Ecoflex 00-50 and the pore-forming agent (PFA)—ethanol (20% vol. relative to the silicone volume)—the foamed silicone is fabricated by addition of the ethanol to the Ecoflex component A (Figure 1a) and stirred manually for 1 min, and then mixed with the component B for 1 min (Figure 1b). The compound was cast into the mold (Figure 1c) manufactured by a 3D printer (Bambu Lab, Shenzhen, China) from acrylonitrile butadiene styrene (ABS). The sample cross-section size is 10 × 10 mm, and the length is 40 mm, fit for the expansion testing.

Curing lasted 3 h at room temperature, then the cured composite specimen was taken from the mold and placed overnight in the heater cabinet (Figure 1d) at 65 °C that is below the PFA (ethanol) boiling point to remove the rest of the PFA from the pores of the composite.

The subsequent rejuvenation procedure [27] (Figure 1f) is used. The rejuvenation is achieved by immersion of the foamed composite in the WL or the solution of SS—benzoic acid in WL (Figure 1e)—allowing its diffusion through the silicone matrix into the pores until the saturation. Different WLs or WL + SS used for the composite rejuvenation do not affect parameters such as pore density, its size and distribution, or the crosslinking of the foamed silicone. However, the use of different PFAs will impact these parameters [25,32]. The rejuvenated content of the WL and WL + SS in the pores is governed by their vapor pressure, pore size, SS concentration in the certain WL, etc. Repeatable rejuvenation of a foamed composite is also possible (up to 100% of its functionality for pure C1–C3 alcohols). The duration of the rejuvenation was not less than 24 h. The detailed preparation procedure is described in Supplementary Materials S1.

The foamed silicone composites (i.e., those having spherical isolated pores) have been demonstrated initially by A. Miriyev et al. using Ecoflex 00-50 silicone and ethanol both as a pore-forming agent and a working liquid [26]. The fabrication of foamed silicone composites was made using ethanol as the PFA [32] at 20% vol. concentration for all the samples containing the sublimation substance.

The SS in our experiments was benzoic acid. WLs were the solutions of the benzoic acid in different alcohols C1–C3: in methanol (56%), ethanol (55%), isopropanol (47%), and propanol (37%). The concentrations shown in brackets were chosen near the solubility limit of benzoic acid in the alcohols used.

For statistics, at least five samples of each type of composite composition were prepared and tested.

### 2.3. Laboratory Equipment

At all stages of the work, a precise analytical balance Shimadzu ATX224 (Kyoto, Japan) and a heating cabinet ShS-80-01 SPU (SKTB-SPU, Smolensk, Russia) for the solvent evaporation were used.

### 2.4. IR Spectra Measurements

The IR spectra of the composites were registered by Tensor 27 IR Spectrometer (Brucker, Rheinstetten, Germany) equipped with a single attenuated total reflectance attachment MIRacle ATR (Pike Technologies, Madison, WI, USA). The attachment was equipped by Diamond/ZnSe performance crystal (4000–550 cm^−1^), and the measurements were made at 2 cm^−1^ resolution using 64 scans.

### 2.5. Compression Test

The static tensile tester Multitest 2.5-XT (Mecmesin Ltd., Slinfold, UK) was used for mechanical testing, and experiments were carried out at room temperature. The compression was conducted in a transverse direction of the prepared samples 10 × 10 × 40 mm^3^. We used an indenter of 5 mm in diameter, positioned it in the center of the sample side surface, and registered the force of the indenter on 2 mm depth. More details of the test procedure are given in Supplementary Materials S3.

### 2.6. Thermal Expansion Measurements, the Device “PARUS”

For the tests of the soft composites having high coefficients of the thermal expansion, the device “PARUS” was used. The composite sample was electrically heated in the PARUS cell of a 10 × 10 mm^2^ cross-section by applying constant power with simultaneous extension control, and the temperatures was raised up enough for the liquid–gas phase transition process in the composite pores effected. A detailed description is given in [32] as well as in Supplementary Materials S2.

**Figure 2:**
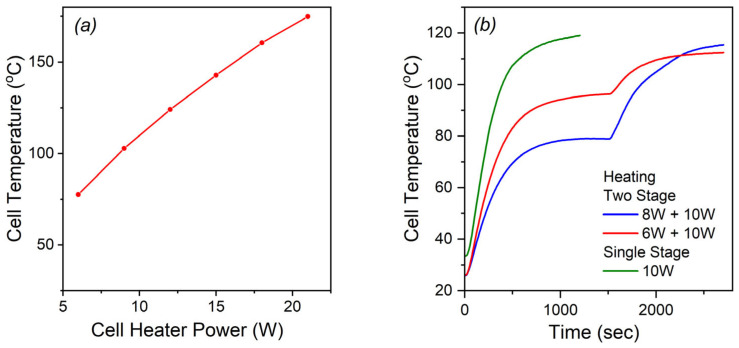
The sample temperature: (**a**) at different cell heater powers; (**b**) during the two-stage heating procedure.

The test for the revealing of SS impact on the strain of a foamed composite was designed to have two stages. At Stage I of the experiment, the composite was heated up to 15–20 °C above the boiling point of the WL to allow its evaporation whilst the sublimation process of SS is rather slow. Then, after 1500 s (25 min), Stage II starts and it lasts 1200 s (20 min)—the temperature increases but it should be significantly lower than the boiling point of the sublimation substance. Figure 2a presents the solid Ecoflex 00-50 sample temperatures that can be reached in the PARUS cell at a different heating power. They were used as references for the heating power selection. The power levels selected are as follows: Stage I heating power for methanol—6 W, for ethanol and isopropanol—8 W; Stage II—benzoic acid sublimation—10 W. For propanol, the two-stage test was not performed because of its rather high boiling point (97 °C) requiring 10 W heating—the same as for Stage II.

The common single-stage actuation test, i.e., close to a real actuation, was always performed at 10 W heating (20 min) in the PARUS device.

The temperature changing for two-stage and single-stage experiments are presented in Figure 2b. At Stage I, the temperature reaches 78 °C at 6 W heating or 97 °C at 8 W heating. During Stage II, 10 W heating rises the temperature to about 120 °C. The experiment duration was 2700 s (45 min). In the single-stage experiment, the temperature also reaches 120 °C.

The data presented in the Section 3 are the averages of 5 experiments, each having a relative standard deviation (%RSD) better than 17%; generally the %RSD is less than 15%. The main sources of the error were the random process of pores formation in the silicone matrix and, to a lesser degree, to the PARUS device measurement accuracy.

## 3. Results

### 3.1. Foamed Composite Filled with the Sublimation Substance

For the first time, we demonstrate that the composite can be rejuvenated not only by pure alcohols of different types, for example, C1–C4 [32], but also by solutions of a solid substance in alcohols.

**Figure 3:**
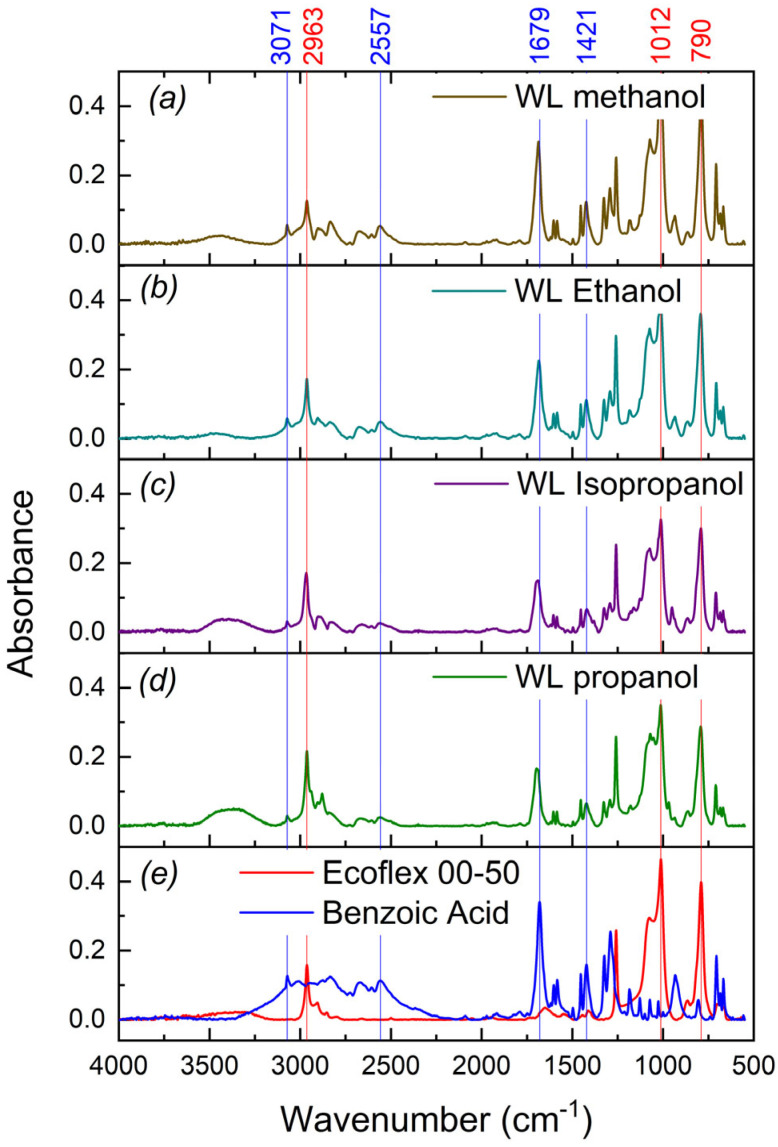
IR spectra: (**a**–**d**) composite rejuvenated by WL ((**a**)—methanol (brown), (**b**)—ethanol (sky blue), (**c**)—isopropanol (magenta), (**d**)—propanol (green)), with benzoic acid, (**e**) Ecoflex 00-50 (red) and benzoic acid (blue).

Figure 3 shows the IR spectra of the inner part of the foamed silicone samples that were rejuvenated in WL + SS. After the procedure with a certain solution, a slice of the sample midpart section was prepared as described below. First, the incision of 2 mm thickness was made round the sample sides. Second, the sample was stretched and a thin midpart was bisected, and immediately after the cut, the IR spectra of the inner sample parts were recorded.

The spectra were measured in the region of 4000–550 cm^−1^. For comparison, in Figure 3a, the spectra with a number of marked peaks are presented for pure Ecoflex 00-50 (2963 cm^−1^ ν^as^ C-H, 2853 cm^−1^ ν^sym^ C-H, 1246 cm^−1^ δ Si-C, 1012 cm^−1^ Si-O) and benzoic acid (3300–2500 cm^−1^ ν O-H). The stretching O-H vibrations overlap the C-H aryl stretching vibrations ν^CH^∼3030 cm^−1^; 1679 cm^−1^ νC=O, 1421 cm^−1^ νC=C, 1260 cm^−1^ νC-O, and 950 cm^−1^ ω O-H. All rejuvenated samples (Figure 3b–e) also have such peaks in their spectra showing the positive rejuvenation result. Also, a broad absorption band around 3350 cm^−1^ indicates the presence of stretching vibrations of hydroxyl groups O-H typical for alcohols.

The IR spectra unambiguously confirm that the composites are rejuvenated with both WL—alcohol and SS—benzoic acid.

### 3.2. Foamed Composite Behavior with the Sublimation Substance in Two-Stage Heating Test

**Figure 4:**
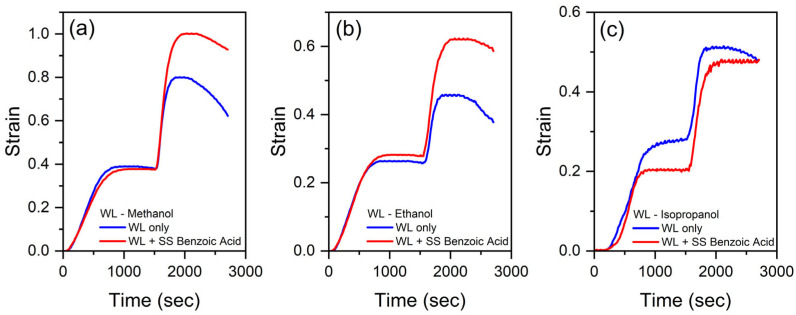
The results of PARUS experiments for rejuvenated foamed Ecoflex 00-50 using different working liquids with benzoic acid as a sublimation substance: working liquid (**a**) methanol, (**b**) ethanol, and (**c**) isopropanol.

The two-stage heating mode of the rejuvenated composite testing was described in Section 2.5. Initially, every composite sample prepared was rejuvenated and further tested with a WL that will be used on the next experiment. Then, after WL + SS rejuvenation, the sample was tested again. Figure 4 shows the results of the experiments for methanol, ethanol, and isopropanol as a WL.

It is clearly seen from Figure 4 that the strains at heating Stage I for the samples rejuvenated by WL only and for the samples rejuvenated using WL with SS are close to each other. However, at stage II, the strains become significantly higher for the samples containing SS.

At Stage I (6 W or 8 W heating), the strains for different WLs do not depend on the presence of SS in the sample, i.e., one can say that the SS sublimation is rather unimportant at low temperatures. The strain for Stage II significantly differs for WL and WL + SS samples. At Stage II (10 W heating), the strain for WL + SS is significantly higher than those for WL only. At the end of the experiment, we find some strain decrease for the samples caused by WL (+SS) evaporation from the composites investigated and, as a consequence, the pores’ pressure decrease makes the strain less. Having in mind that methanol diffusion through a silicone is rather higher [34], the strain decrease also begins earlier for the methanol samples than for the ethanol ones. Therefore, the composite actuation will last for a certain time, which depends on the WL used.

Table 1 presents the averaged strains (ε=Δl/l) at Stages I and II and calculated ratios revealing the measure of SS—benzoic acid influence on the actuation of thermomechanical actuators. The results were obtained for ethanol, methanol, and isopropanol as WLs.

In all cases investigated, the ratios for the composites with filled WL only are less than those for the corresponding composites filled with WL + SS solutions. In average, analysis of the data in Table 1 shows that in the two-stage heating mode, the ratio is 18% higher, averaged over all the WL data as well as the Stage II strain that is higher for WL + SS, except WL—isopropanol that demonstrates almost equal strains.

### 3.3. Foamed Composites Behavior with the Sublimation Substance at a Single-Stage 10 W Heating

The single-stage heating is a common type of actuation of the thermomechanical composites in practice. In view of this, we repeated our experiments in a single-stage mode using 10 W heating.

In the work [27], it was proved that multiple sample rejuvenation is possible after the actuation without a loss of performance in the case of WL ethanol, and the same was shown for the other alcohols C1–C4 as well [32]. Moreover, in the open-air conditions, C1–C4 filled composites are able to perform several actuations without a rejuvenation procedure [32]. However, the mechanical properties may deteriorate due to the reactivity of benzoic acid, which, at elevated temperatures, can react with the alcohol, forming corresponding alkyl benzoate. This can lead to partial swelling of the siloxane network.

**Figure 5:**
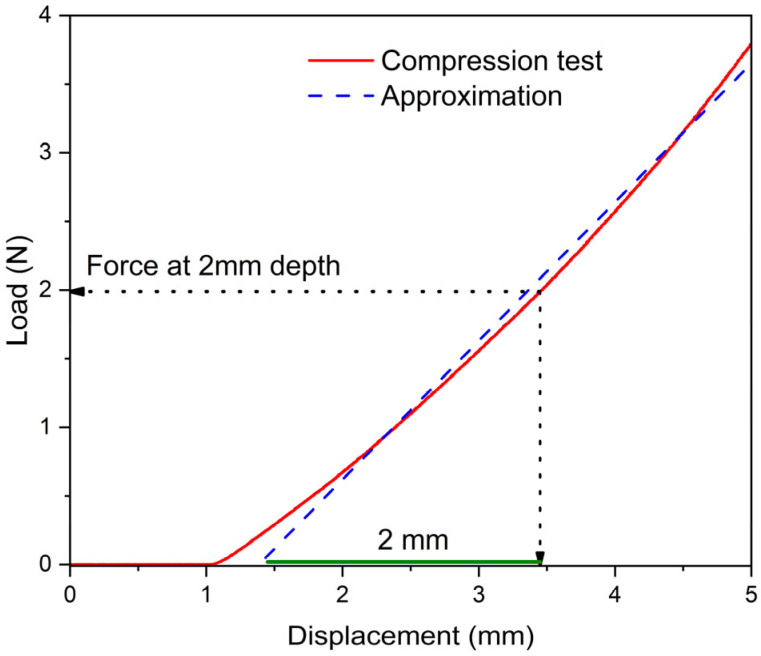
Typical force–indenter depth curve in the compression test. The sample is isopropanol + benzoic acid before actuation. The indenter displacement in the sample is marked in green.

To find the degree of mechanical property degradation, we made the compression tests of our samples after different types of the actuation. The compression was conducted as it is described in Section 2.5. At least two sides of each sample were examined, and results were averaged. The test was similar to the Shore hardness test, which, unfortunately, cannot be applied to our samples because of the foamed structure of the composites. The ratios of measured forces before and after of the single-stage 10 W heating was calculated and is summarized in Table 2, which contains the data obtained for C1–C3 WL only and WL + SS. Figure 5 shows a typical force–indenter displacement curve, as the example chosen is the isopropanol + benzoic acid composite. The 2 mm distance, where the indenter deeps in the sample side, is marked on Figure 5 in green. More force–indenter displacement curves can be found in the Supplementary Materials (Figure S3).

One can find that mechanical properties of the samples did not change significantly after WL-only tests, but after WL + SS tests, the composites lose their rigidity. Namely, this fact also allows us to call our actuators one-off or disposable actuators.

**Figure 6:**
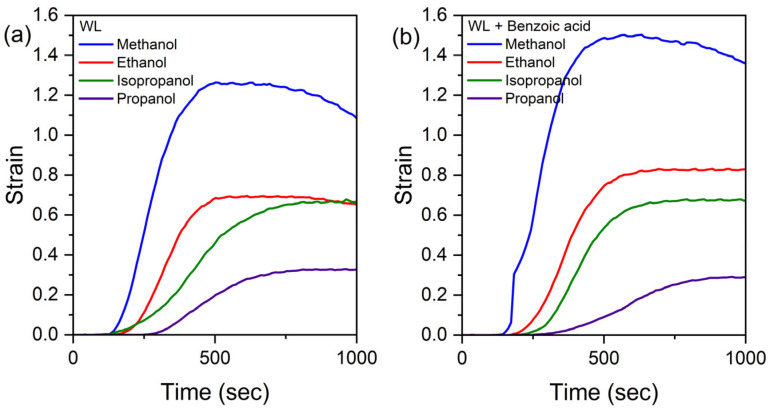
The stress–time experiment in single-stage heating mode rejuvenated with different WLs (shown in the figure) for (**a**) WL only; (**b**) WL + SS benzoic acid.

Typical results of the strain vs. time experiment in single-stage heating mode are shown in Figure 6. Results are presented in the same scale for composites rejuvenated with WL only and WL + SS benzoic acid.

Averaged data for the maximal achieved strains (ε=Δl/l) in one-stage heating mode are given in the Table 3, where the results of testing of alcohols C1–C3 as WLs and benzoic acid as SS are given.

The data in Table 3 clearly show that the SS influence is dissimilar for different alcohols used as WLs. In the case of methanol, the strain is larger than the strain for ethanol, both demonstrating ca. 20% growth. However, isopropanol and propanol strains for WL + SS are close or even less than 1. In Table 1 for isopropanol, one can find similar behavior: Stage II strain for WL + SS is a bit less than those for WL only. Such behavior could have many reasons, for instance, the changes in benzoic acid solubility in certain alcohol and/or the diffusion coefficients variations. Both are higher for methanol and ethanol than for C3 propanols.

## 4. Discussion

In this work, it was shown for the first time that the strain of foamed silicone composite actuators initially employing liquid–gas phase transition can be extended by the addition of the sublimation substance power to the composite rejuvenation procedure. We have used benzoic acid as a sublimation substance.

Using FTIR spectroscopy, it was shown that the sublimation substance—benzoic acid—can penetrate (diffuse) into the composite pores along with the working fluid—C1–C3 alcohols—and, as a result, enhance the activation strain.

The proposed two-stage heating mode (or two-stage activation) allowed us to estimate the contribution of the sublimation substance power to the overall activation of the foamed composite. In the two-stage heating mode, the first stage causes a liquid–gas phase transition in the working fluid (C1–C3 alcohols), while the second stage allows us to reveal the changes caused by the introduced sublimation substance power. In the usual single-stage heating mode, which happens in practice, an increase in the strain was also observed.

The chemical nature of benzoic acid deteriorates mechanical properties of the composite—a decrease in elasticity after the activation (heating) process was observed. To evaluate the elasticity changes, a method similar to the Shore hardness measurement was proposed, applicable to highly porous and, therefore, extremely soft composites, where a Shore-type indenter cannot be employed.

The type of strain–time (temperature) curves during activation—namely, constant (and then slightly decreasing) strain upon reaching a given activation temperature—precludes a direct link between the decrease in composite elasticity and the increase in activation strain, since the strain at a constant activation temperature should increase. However, a possible separation of the effects of these phenomena on the actuation strain—sublimation power and elasticity reduction—were not investigated within the scope of this work.

It should also be noted that the tests conducted are performed in open-air conditions and during the actuation, both the working fluid (alcohols) and the sublimation agent (benzoic acid) leave the composite body, turning it into a disposable actuator.

## 5. Conclusions

The results reported show for the first time that it is possible to rejuvenate the foamed composite initially employing liquid–gas phase transition by the sublimation substance solution in the C1–C3 alcohols, and in this work, benzoic acid was used for this purpose.

The effect of the benzoic acid addition was revealed using the two-stage heating mode that enhances the composite strain ratios for all tested alcohols by approx. 18%. The strains values for methanol and ethanol as working liquids also become higher by about 20%, but for isopropanol, they are left nearly unchanged.

Single-stage heating, which is a more realistic application for the actuators, was used for testing as well. It demonstrated that for methanol and ethanol, the strains are higher on 23% and 17%, respectively, but for C3 alcohols (isopropanol and propanol) the sublimation power influence is low or even negative. Looking at ecological issues of such type of composite rejuvenation, it is worth mentioning that the combination of ethanol + benzoic acid (E210) is the most suitable, but, for sure, it depends on the real application conditions.

Thermomechanical actuators based on the composites employing a liquid–gas phase transition with a sublimation substance are promising for use in many different protecting applications, like safety valves or thermo-actuated switches, disposal soft but powerful grips or jaws, etc. On the analogy of soft robotics-based and soft actuators, such units can be named “soft safetics” units. The results obtained in this work pave the way for future powerful multipurpose safety appliances.

## Figures and Tables

**Table 1:** The strains for two-stage heating for the foamed silicone composites rejuvenated by WL only and WL + SS.

	WL Only			WL + SS (Benzoic Acid)		
WL	Stage I	Stage II	$\frac{\mathrm{Stage}\;\mathrm{II}}{\mathrm{Stage}\;I}$	Stage I	Stage II	$\frac{\mathrm{Stage}\;\mathrm{II}}{\mathrm{Stage}\;I}$
Methanol	0.45	0.85	1.89	0.47	1.06	2.23
Ethanol	0.30	0.51	1.69	0.31	0.63	2.02
Isopropanol	0.27	0.47	1.75	0.22	0.45	2.03

**Table 2:** The ratio of forces in compression tests (indenter at 2 mm depth) for the samples after one-stage 10 W heating tests for different methods of the rejuvenation (the %RSD for presented data is less than 15%).

WL	Before Test	After WL-Only Test		After WL + SS Test	
	Force (N) @ 2 mm	Force (N) @ 2 mm	Ratio	Force (N) @ 2 mm	Ratio
Methanol	1.97	1.95	0.99	0.67	0.34
Ethanol		1.87	0.95	0.89	0.45
Isopropanol		1.93	0.98	0.98	0.50
Propanol		2.01	1.02	1.05	0.53

**Table 3:** The composite strain at a single-stage heating mode for the composite rejuvenated with alcohols C1–C3 as WL only and WL + SS benzoic acid.

WL	WL Only	WL + SS	Ratio
Methanol	1.18	1.45	1.23
Ethanol	0.71	0.83	1.17
Isopropanol	0.73	0.68	0.93
Propanol	0.34	0.31	0.91

## Data Availability

The data that support the findings of this study are available from the corresponding author upon reasonable request.

## Supplementary Materials

The following supporting information can be downloaded at https://www.mdpi.com/article/10.3390/polym17223032/s1; Preparation of foamed silicone with sublimation substance; Figure S1: Preparation of foamed silicone; Detailed description of the PARUS device; Figure S2: The PARUS device; Table S1: Working liquids: boiling temperature, molecular weight, cell heater power; Detailed description of the indenter experiment data treatment; Figure S3: The corrected displacement-load data for the indenter test.

## References

[1] Tang X., Li H., Ma T., Yang Y., Luo J., Wang H., Jiang P. (2022). A Review of Soft Actuator Motion: Actuation, Design, Manufacturing and Applications. Actuators.

[2] Narvaez D., Newell B. (2025). A Review of Electroactive Polymers in Sensing and Actuator Applications. Actuators.

[3] Ahn J., Gu J., Choi J., Han C., Jeong Y., Park J., Cho S., Oh Y.S., Jeong J.H., Amjadi M. (2022). A Review of Recent Advances in Electrically Driven Polymer—Based Flexible Actuators: Smart Materials, Structures, and Their Applications. Adv. Mater. Technol..

[4] Agrawal R., Koteswarapavan C., Kaushik N., Matre P. (2019). Smart Actuators for Innovative Biomedical Applications: An Interactive Overview. Applied Microbiology and Bioengineering.

[5] Raman R. (2024). Biofabrication of Living Actuators. Annu. Rev. Biomed. Eng..

[6] Ghazaryan G., Khmelnitskaia A., Bezsudnov I., Kalinina A., Agina E., Ponomarenko S. (2023). A Concise Guide to Silicone-Based Spring-Roll Actuator Assembly. Polymers.

[7] Hao M., Wang Y., Zhu Z., He Q., Zhu D., Luo M. (2019). A Compact Review of IPMC as Soft Actuator and Sensor: Current Trends, Challenges, and Potential Solutions From Our Recent Work. Front. Robot. AI.

[8] Zhang H., Lin Z., Hu Y., Ma S., Liang Y., Ren L., Ren L. (2023). Low—Voltage Driven Ionic Polymer—Metal Composite Actuators: Structures, Materials, and Applications. Adv. Sci..

[9] Dayyoub T., Zadorozhnyy M., Ladokhin D.G., Askerov E., Filippova K.V., Iudina L.D., Iushina E., Telyshev D.V., Maksimkin A. (2025). Ionic Electroactive Polymers as Renewable Materials and Their Actuators: A Review. J. Renew. Mater..

[10] Wang H., Wang Z., Yang J., Xu C., Zhang Q., Peng Z. (2018). Ionic Gels and Their Applications in Stretchable Electronics. Macromol. Rapid Commun..

[11] Fang C., Lu Z., Hu C., Gao Y., Zhu J., Hu W. (2024). Research progress on conductive polymer actuators: Mechanisms, performance improvement, and applications. Mater. Today Commun..

[12] Hu F., Xue Y., Xu J., Lu B. (2019). PEDOT-Based Conducting Polymer Actuators. Front. Robot. AI.

[13] Li X., Wang Y., Xu M., Shi Y., Wang H., Yang X., Ying H., Zhang Q. (2021). Polymer electrets and their applications. J. Appl. Polym. Sci..

[14] Saxena P., Shukla P. (2020). A Review of Polymer Electrets and Their Applications. Mater. Perform. Charact..

[15] Bae J.-H., Chang S.-H. (2019). PVDF-based ferroelectric polymers and dielectric elastomers for sensor and actuator applications: A review. Funct. Compos. Struct..

[16] Kumar V., Babu T., Tiwari B. (2021). A literature review of the research on ferroelectric polymers. J. Phys. Conf. Ser..

[17] Ince J., Prasad K., Subhani K., Duffy A., Salim N. (2024). Liquid crystalline elastomers as artificial muscles and flexible actuators for robotics/hybrid engineered machinery. Adv. Compos. Hybrid Mater..

[18] Jiang Z.-C., Liu Q., Xiao Y.-Y., Zhao Y. (2024). Liquid crystal elastomers for actuation: A perspective on structure-property-function relation. Prog. Polym. Sci..

[19] Bezsudnov I.V., Khmelnitskaia A.G., Kalinina A.A., Ponomarenko S.A. (2023). Dielectric elastomer actuators: Materials and design. Russ. Chem. Rev..

[20] Prabhakar P., Sahu D., Sahu R.K., Bessudnov I., Ponomarenko S. (2025). Electromechanical Characterization of Dielectric Elastomer Actuators Under Static and Dynamic Electrical Loading for Artificial Muscles. Polym. Eng. Sci..

[21] Wang Y., Ma X., Jiang Y., Zang W., Cao P., Tian M., Ning N., Zhang L. (2022). Dielectric elastomer actuators for artificial muscles: A comprehensive review of soft robot explorations. Resour. Chem. Mater..

[22] Zhang Q., Yu W., Zhao J., Meng C., Guo S. (2025). A Review of the Applications and Challenges of Dielectric Elastomer Actuators in Soft Robotics. Machines.

[23] Ding Z. (2012). Shape Memory Hybrids: Mechanism and Design for Tailored Properties. Ph.D. Thesis.

[24] Lipton J.I., Angle S., Banai R.E., Peretz E., Lipson H. (2016). Electrically Actuated Hydraulic Solids. Adv. Eng. Mater..

[25] Miriyev A., Caires G., Lipson H. (2018). Functional properties of silicone/ethanol soft-actuator composites. Mater. Des..

[26] Miriyev A., Stack K., Lipson H. (2017). Soft material for soft actuators. Nat. Commun..

[27] Miriyev A., Trujillo C., Caires G., Lipson H. (2018). Rejuvenation of soft material-actuator. MRS Commun..

[28] Bilodeau R.A., Miriyev A., Lipson H., Kramer-Bottiglio R. All-soft material system for strong soft actuators. Proceedings of the 2018 IEEE International Conference on Soft Robotics (RoboSoft).

[29] Cartolano M., Xia B., Miriyev A., Lipson H. (2019). Conductive Fabric Heaters for Heat-Activated Soft Actuators. Actuators.

[30] Kaplon T., Lindner T., Wyrwal D. Induction heating for a silicone/ethanol composite actuator. Proceedings of the 2020 International Conference Mechatronic Systems and Materials (MSM).

[31] Xia B., Miriyev A., Trujillo C., Chen N., Cartolano M., Vartak S., Lipson H. (2020). Improving the Actuation Speed and Multi-Cyclic Actuation Characteristics of Silicone/Ethanol Soft Actuators. Actuators.

[32] Bezsudnov I., Khmelnitskaia A., Kalinina A., Monakhova K., Ponomarenko S. (2025). Liquid–Gas Phase Transition Actuator: Rejuvenation Procedure Extended and Open-Air Performance. Polymers.

[33] Miriyev A., Xia B., Joseph J.C., Lipson H. (2019). Additive Manufacturing of Silicone Composites for Soft Actuation. 3D Print. Addit. Manuf..

[34] Ravishankar H., Dessì P., Trudu S., Asunis F., Lens P.N.L. (2021). Silicone membrane contactor for selective volatile fatty acid and alcohol separation. Process Saf. Environ. Prot..

