# Supplementary material for: Disposable Foamed Silicone Composite Actuator Powered by Sublimation
Source: Polymers (Basel). 2025 Nov 15;17(22):3032. doi: 10.3390/polym17223032 (PMC12656423; doi:10.3390/polym17223032)
Supplement: Supplementary file 1 [file polymers-17-03032-s001.zip › polymers-3960996-supplementary.pdf]

Supplementary Material

Disposable Foamed Silicone Composite Actuator Powered by Sublimation

Igor Bezsudnov, Alina Khmelnitskaia, Aleksandra Kalinina, Sergey Ponomarenko

Enikolopov Institute of Synthetic Polymeric Materials of Russian Academy of Sciences, Profsoyuznaya Str. 70, 117393 Moscow, Russia; bezsudnov_iv@ispm.ru (I.B.); alina.khmelnitskaya@ispm.ru (A.Kh.); kalinina@ispm.ru (A.K.); ponomarenko@ispm.ru (S.P.)

SM1. Preparation of a foamed silicone composite with a sublimation substance

The Ecoflex 00-50 components A and B are to be mixed at the 1:1 ratio (Figure S1 (a), Supplementary Material). To fabricate the foamed composite material, the silicone component A was manually stirred for 1 min with ethanol as a PFA (pore forming agent) in the amount to achieve targed vol.% relative to the full A+B composite volume (Figure S1 (b)), then manually mixed with the component B for 1 min (Figure S1 (c)). The mixture was cast into molds made by a 3D printer using ABS (acrylonitrile butadiene styrene) (Figure S1 (d)). Two types of molds were used: 20 mm dia. mold (for porosity investigation) and 10 × 10 mm section mold with the 40 mm length for use in the PARUS device (see below) for expansion testing.

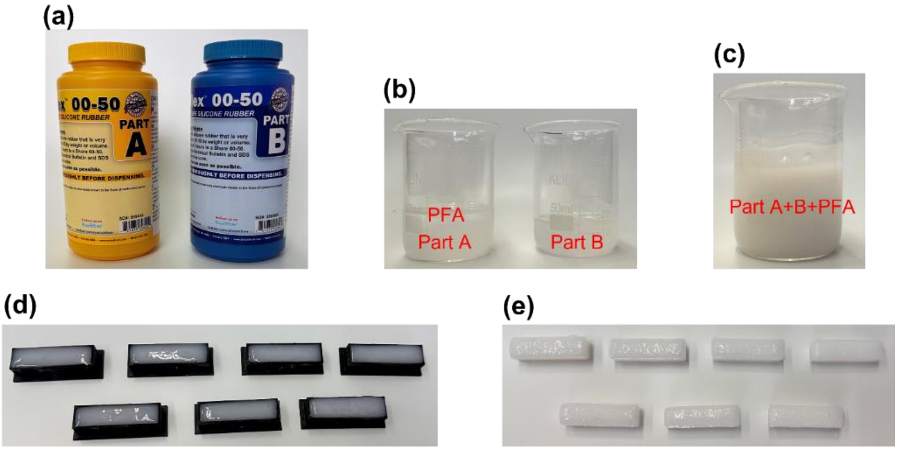

Figure. S1. Preparation of a foamed silicone composite. (a) Two-component PDMS Ecoflex 00-50 (Smooth-On, PA, USA); (b) Component A in ethanol and component B; (c) Mixture of the components; (d) Casting in molds; (e) Ready-made silicone composite.

It takes at least three hours to cure the cast material at room temperature. Once the silicone had cured and the ready specimen was removed from the mold, it was processed overnight in a heater cabinet at the temperature of 65 °C that is ca. 13 °C less than the boiling point of

ethanol used as the PFA to initially remove the preparation solvent from the pores of the composite (Figure S1 (e)).

SM2. Detailed description of the PARUS device

The PARUS device and its cell are shown in Figure S2. The device is controlled by the Arduino Nano v.3 microcontroller, the block diagram is shown in Figure S2c. The control program was created in the Arduino IDE v1.8.19 development environment. The host computer is connected to the instrument via the USB port; in the manual mode, the PARUS device is controlled via a monitor of the connected serial port using one-character commands, in the automatic mode – by the specially developed software running in the MS Windows™ environment.

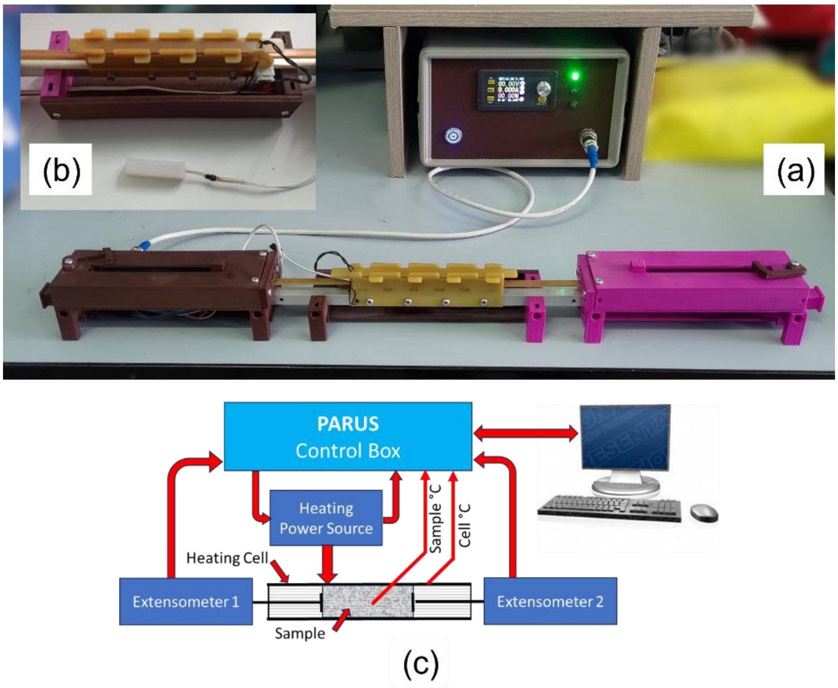

Figure S2. The PARUS device. (**a**) A photo of the front view of the device: control block with the heating power source and the extensometer, (**b**) the cell with a sample installed (on the top) and the sample with a temperature sensor inserted (on the bottom), (**c**) the PARUS device block diagram.

The PARUS device employs an external heating for a sample placed in the cell, see Figure S2b. The cell has a square cross-section of 10×10mm. The walls are made of a glass-reinforced epoxy material FR-4 usually used for PCB manufacturing, with a thickness of 1.75 mm, a copper thickness of 35 µm, and a maximum operating temperature of 175 °C, the copper side is inside the cell. The heater is a copper meander etched on the inner surfaces of the cell walls, the width of the conductor is 0.25 mm, and the total length of the conductor is about 7 m. All the composite samples have a cross-section of 10×10 mm and a length of 25 - 50 mm.

To power the cell heater, a cascade of two power sources is used: the unregulated power supply model LRS-100 (Mean Well, Taiwan) with an output voltage of 48V, current 2.3A, and the microprocessor-controlled power supply model DSP5005 (Gaqqee, China), the output voltage range

is 0-45V and the current up to 5A, the accuracy of voltage/current setting is 0.01V / 0.001A. In this work we perform heating using a constant power, the maximal power supplied to the cell heater can be up to 30W. Every 15 sec the supplied power is checked and corrected.

Two types of temperature measurements were realized. The temperature of the heated internal wall surface of the cell, i.e., the temperature of the sample surface, is measured by the resistance of the cell copper heater. The actual thermal coefficient is specified in the code. The temperature measurement accuracy is ±1.5 °C. The PARUS device also measures the temperature inside the sample volume, it uses a temperature sensor model NTC-100 with a measurement range of -50 ±260 °C, resistance at 25 °C 100kΩ ± 1%, and the temperature measurement accuracy is better than 0.5 °C. This sensor was placed in the center of the composite sample through a puncture made with a thin awl to the middle of the sample (Figure S2b), where the composite sample is shown with a temperature sensor inserted.

The sample elongation sensors (extensometers) measuring a distance of up to 90 mm were manufactured for this device based on a pre-calibrated sliding variable resistor with a nominal value of 10 kΩ, length of 100 mm, measurement accuracy of 0.2 mm for whole measurement range. The instrument uses two identical extensometers. When measuring the resistance of extensometers, a highly stable ADC ADS1115 with an internal voltage reference is used. Three LED indicators and a buzzer indicate the status of the PARUS device.

Figure 2a shows the maximum temperature of the Ecoflex 00-50 solid silicone sample in the PARUS device cell at different heating power. The data make it possible to choose heating power for the foamed silicone samples.

Table S1 presents working liquids used in this work together with their boiling temperature, and chosen cell power for 1-st stage of two-stage heating mode, 2-nd stage is always 10W heating.

Table S1. Working liquids: boiling temperature, molecular weight, cell heater power.

Working Liquid	Boling Temp. (BT)	Molecular weight (MM)	Heating power (W)
Methanol	65	32.0	6.0
Ethanol	78	46.1	8.0
Isopropanol	82	60.1	8.0
Propanol	97	60.1	10.0

SM3. Detailed description indenter experiment data treatment

To allow the correct comparison of 2mm depth indenter force test we used the following two-step procedure. 1. It was built the linear approximation of the displacement-load dependency after the indenter touched the sample; 2. The displacement-load data were shifted in the way the above linear approximation crosses the displacement axis at zero value.

The linear approximation is built using the middle third part of the displacement-load data which the values that are over 0.1N. The calculated shift and slope are further used to find the displacement correction.

Figure S3 shows the displacement-load data corrected using above procedure. Each graph in the figure presents corrected indenter test data before the experiment and after the test with WL + SS (solid lines) and appropriate linear approximations (dashed lines) crossing at zero value the displacement axis allowing to find easily the force at 2mm (dashed reference line) indenter depth as well as the radio of forces at this depth (see Table 2).

The data for WL only experiments are not presented because they are very close to the before experiment data.

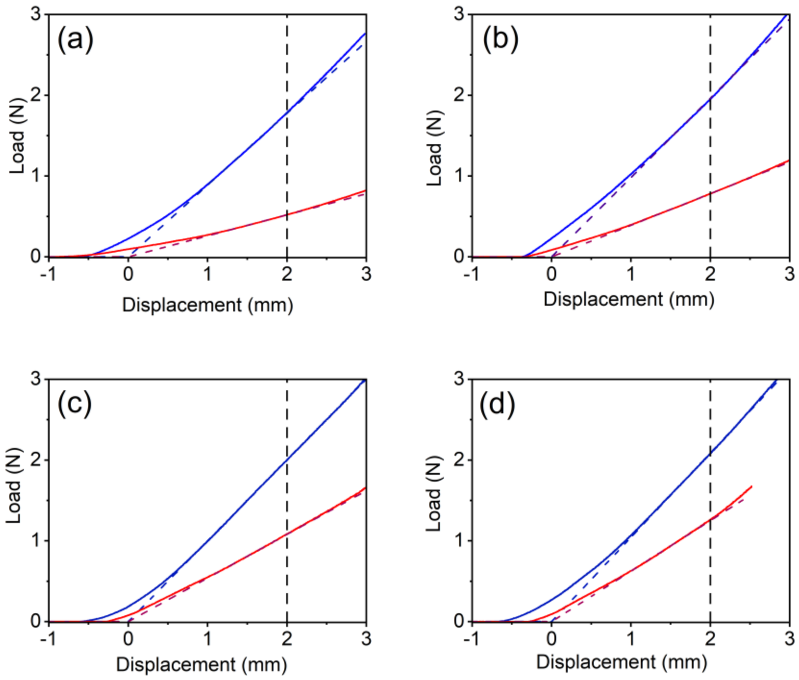

Figure S3. The corrected displacement-load data for the indenter test for different WL: (a) methanol, (b) ethanol, (c) isopropanol, (d) propanol. Solid lines – corrected experiment data: blue – before the experiment, red – after WL + SS Benzoic acid experiment. Dashed lines – linear approximation of above. Black dashed reference line at 2 mm actual indenter depth.
